# Plasticizer Mixing Improved Regenerated Cellulose Films as an Alternative to Plastics

**DOI:** 10.1021/acssuschemeng.5c00491

**Published:** 2025-07-07

**Authors:** Pauliina Ahokas, Vesa Kunnari, Johanna Majoinen, Ali Harlin, Mikko Mäkelä

**Affiliations:** 3259 VTT Technical Research Centre of Finland Ltd., Tietotie 4E, Espoo 02044, Finland

**Keywords:** cellulose films, crystallinity, mixture design, permeability, regression modeling, tensile strength

## Abstract

Plasticizers are widely used to improve the elasticity of regenerated cellulose films, but academic research has thus far ignored the possibility of plasticizer mixing. We studied the effects of glycerol, sorbitol, and maltitol on film properties based on a systematic design for mixture experiments and determined regression models to correlate the mixture composition with film properties. Our results showed that plasticizer mixing enabled us to control film tensile strength and water vapor and oxygen permeabilities, which are key indicators for evaluating film efficacy in barrier packaging applications. Our films showed lower water vapor and oxygen permeabilities than commercial uncoated cellophane films and could potentially provide a renewable alternative to conventional polyolefin films with further improvements. These results are important and they indicate that plasticizer mixing provides a novel and simple methodology to improve regenerated cellulose films to meet the increasing demand for alternatives to conventional plastics in packaging and other applications.

## Graphical abstract

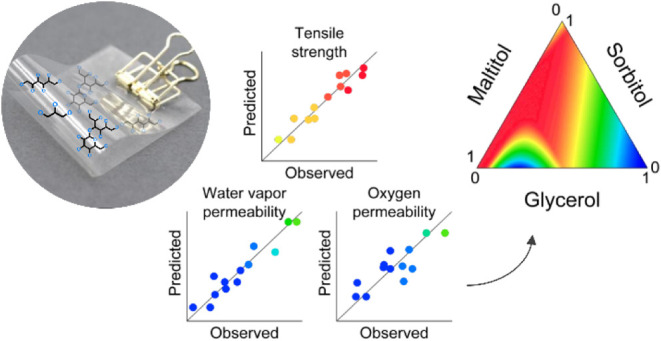

## Introduction

Petroleum-based plastics and plastic films are widely used in packaging applications. The global production of plastics increased to 460 million tonnes in 2019 and 31% of these plastics were used for packaging.^1^ An important application for plastics in the packaging segment is barrier packaging. Flexible barrier films, for example, are generally produced from low-density polyethylene or polypropylene with excellent moisture barrier and chemical resistance properties to ensure a satisfactory shelf life in food packaging applications. Both of these plastic types have typically low barrier performance against oxygen and carbon dioxide, which can be improved by adding poly­(vinyl alcohol), ethylene vinyl alcohol, polyvinylidene chloride, or aluminum oxide coatings as additional layers.^2–4^ None of these common alternatives are, however, biodegradable, and they are often difficult to recycle. Multilayer films for fresh food packaging can contain up to seven individual layers and are particularly difficult to recycle when they contain halogenated compounds, metal foils, or metallized films as functional layers.^5,6^ Recycling reduces waste generated by the packaging industry, which has an average product lifetime of 0.5 years from production to disposal compared to 5 and 35 years of the textile and construction industries, respectively.^7^ In 2019, low-density polyethylene and polypropylene from packaging formed 20% of plastic waste, which is projected to reach 1.2 billion tonnes by 2060.^8^ Only 17% of these wastes are estimated to be recycled, and the rest will be incinerated, landfilled, or mismanaged.^9^ These challenges have been acknowledged by the European Commission, which proposed further changes to Directive 94/62/EC on packaging and packaging waste in 2022, aiming to prevent waste and to ensure that all packaging is cost-effective, reusable, or recyclable by 2030.^10^

Regenerated cellulose films are a potential alternative to petroleum-based plastics in barrier packaging. Cellulose is not naturally thermoplastic but can be regenerated into different forms, such as thin films or coatings, through dissolution and precipitation. Dissolution breaks down the supramolecular structure of native cellulose I, and precipitation reorients the scattered polymer chains in opposite directions to form the regenerated cellulose II polymorph.^11^ Several dissolution and precipitation chemistries have been introduced in the past century. Xanthation and alkali dissolution are used to produce viscose yarn, which is the largest regenerated cellulose application with a 90% market share.^12^ Xanthation is, however, connected to serious environmental and occupational risks due to the use of carbon disulfide and the formation of sulfur-containing byproducts.^13^ A promising alternative is to mechanically modify cellulose while pretreating it with enzymes. Moist dissolving-grade pulp can be exposed to a tailored enzyme-cocktail and ground to improve enzyme accessibility by increasing fiber porosity.^14^ This mechano-enzymatic pretreatment decreases the degree of polymerization of cellulose and increases porosity, with both improving dissolution into aqueous sodium zincate solution.^14–17^

Enzymatic mechanical pretreatment and sodium zincate dissolution can be used to modify cellulose into biodegradable films for packaging applications. Regenerated cellulose films, however, have generally low elasticity and low-to-moderate barrier properties against water vapor and oxygen. Mechanical film properties are commonly improved with plasticizers, which are usually low-molecular-weight compounds used to increase film elasticity, and impregnated into the film structure by diffusion.^18^ Phthalates, for example, are plasticizers used in food casings and personal care items primarily prepared from synthetic polymers despite the increasing information on their negative impact on human health.^19,20^ Studies on alternative biobased plasticizers have mainly focused on epoxidized oils, cellulose derivatives, and polyols such as glycerol and its derivatives.^18,21–23^ Considerably fewer studies have combined different plasticizers^24–27^ and we found no academic studies focusing specifically on plasticizer mixing.

Our objective was to improve the properties of regenerated cellulose films by focusing on plasticizer mixing. We hypothesized that mixing low-molecular-weight polyhydroxylic compounds with different numbers of hydroxyl groups could enable us to control the mechanical and barrier properties of cellulose films based on mixture composition. Our hypothesis was based on the work of Fernández-Santos et al.^28^ who reported that the barrier properties of nanocellulose films improved with an increasing number of hydroxyl groups in the plasticizer. Thus, we studied the effects of glycerol, sorbitol, and maltitol on film properties using a systematic design for mixture experiments and determined regression models to correlate the mixture composition with film properties. We focused specifically on tensile strength and water vapor and oxygen permeabilities as they are considered key indicators of film efficacy for barrier applications.^28,29^ Our results are important to understand the effects of plasticizers on film properties and to develop new plasticizer mixing strategies to improve film performance. These developments can improve the competitiveness of regenerated cellulose films against their petroleum-based counterparts in packaging and other applications.

## Experimental Section

### Design of Experiments

The plasticizer experiments were designed according to a simplex centroid design originally reported by Scheffe.^30^ The original mixture design was augmented with additional axial points, which contained mixtures of all three plasticizers (Figure 2a). These experiments were added to improve the predictive ability of the experiments specifically for ternary mixtures. We also replicated the glycerol, sorbitol, and maltitol experiments, which generated 13 plasticizer mixture experiments (Table S1). Nonplasticized cellulose films were used as control samples.

### Film Preparation and Characterization

Dissolving-grade softwood sulfite pulp purchased from Domsjö Fabriker AB was used for preparing the films. The pulp was disintegrated in deionized water and spin-dried to 33 wt % dry matter content. The spin-dried pulp was then pretreated by enzymatic twin-screw extrusion. An enzyme mixture FiberCare R purchased from Novozymes was added to the pulp while feeding it through a twin-screw extruder for 1.5 min at 50–52 °C. The pulp was left to age, and the enzymes were then inactivated by immersing the pulp in aqueous NaOH (pH 11), followed by washing with water. The intrinsic viscosity of the pulp was measured by using a standard procedure, and the viscometric average degree of polymerization was determined with Mark–Houwink’s equation (240 mL·g^–1^ and 310, respectively).^31,32^ The pretreated pulp was dissolved in sodium zincate by a two-stage direct dissolution procedure, and the mixture was −3 °C at the end of the dissolution. The obtained dissolved cellulose solution was an aqueous mixture of 6.7 wt % pulp, 8 wt % NaOH, and 1.6 wt % ZnO. This solution was deaerated at 120 mbar overnight and casted on glass plates. The cast films were precipitated in an aqueous solution of 10 wt % H_2_SO_4_ and 10 wt % Na_2_SO_4_ and washed with tap water. Ten visually homogeneous films were then plasticized in an aqueous 5 wt % plasticizer solution for 60 s. The plasticized films were dried on jigs to prevent shrinkage. All films were conditioned at 23 °C and 50% relative humidity for at least 24 h before further analysis.

Tensile properties of the films were determined with a Lloyd LS5 testing instrument (AMETEK measurement and calibration technologies, USA) by using a 100 N load cell. The films were randomly selected and cut into 15 mm × 80 mm long strips, and at least six different film strips were measured for each experimental point. The initial gauge length was 50 mm, and gauges were pulled apart with an extension rate of 5 mm·min^–1^. Young’s modulus was defined as a stress–strain slope of >80% of the two-point slope maxima. Water vapor permeation (WVP) was measured with a PERMATRAN-W 3/34 analyzer (AMETEK MOCON, USA). Oxygen permeation (OP) was measured with an OX-TRAN 2/22H Permeation Analyzer (AMETEK MOCON, USA). For both WVP and OP measurements, the temperature was set at 23 °C in 50% relative humidity, and measurements were carried out by using a cell area of 5 cm^2^.

Wide-angle X-ray scattering (WAXS) spectra were recorded with a Xenocs Xeuss 3.0 SAXS/WAXS system (Xenocs SAS, Grenoble, France). The system included a microfocus X-ray source (sealed tube, operating at 50 kV and 0.6 mA) with a Cu target and a multilayer mirror, yielding a parallel beam with a nominal wavelength of 1.542 Å (combined Cu K-α1 and Cu K-α2 characteristic radiation). The beam size at the sample was set to 0.4 mm. A Kapton film was used as a sample holder, and its scattering signal was normalized and deducted from the measurement data. An area detector (Eiger2 R 1M, Dectris AG, Switzerland) recorded the data. The distance between the sample and the detector was calibrated by measuring the diffraction of the known standard sample LaB6. The total crystallinity index was determined based on the maximum peak intensities of the main crystalline planes (*I*_110_) at around 20° and the minima (*I*_AM_) between the 1–10 and 110 peaks at around 16° ^33–35^ according to the method developed by Segal et al.^36^ (eq 1):

1 $$\mathrm{Total crystallinity index}=\left(\frac{I_{110}-I_{\mathrm{AM}}}{I_{110}}\right)$$

The crystallite size (τ) of the main crystalline plane *I*_110_ at around 20° was calculated by using the Scherrer equation^37,38^ (eq 2):

2 $$\tau =\frac{\lambda K}{\beta \cos\theta }$$

where *K* denotes a crystal shape-dependent constant;^37^ λ is the X-ray wavelength of the incident beam in the diffraction experiment (0.1542); β is the full peak width at half of its maximum; and θ is the Bragg’s angle, i.e., the position of the peak. Full peak width at half of its maximum was determined with nonlinear Gaussian curve fitting in OriginPro 2024 (OriginLab Corp.).

### Empirical Models

Correlations across plasticizer composition and film properties were determined with principal components analysis. The data were compiled in a matrix, and the matrix columns were normalized to unit variance and zero mean to compare variables given in different units. Correlations across the normalized variables were evaluated with the principal component loadings based on the general model equation (eq 3):

3 $$X=\overset{n}{\underset{i=1}{\sum }}t^{T}p+E_{n}$$

where X denotes the normalized and mean-centered data matrix, t denotes the orthogonal score vectors, p denotes the orthonormal loadings vectors, and E_*n*_ denotes a residual matrix after *n* components. The first loadings vector was determined as the set of weights that maximized the variation explained by the corresponding scores^39^ (eq 4):

4 $$\arg\underset{∥w∥=1}{\max}\left(t^{T}t\right)$$

where *w* denoted the component weights, and the loadings of subsequent principal components were set to be orthogonal to the previous ones. The principal components thus formed a new coordinate system that described the main correlations across the plasticizer mixture composition and film properties.

Plasticizer composition was then used to estimate individual film properties with multiple linear regression. Tensile strength and water vapor and oxygen permeabilities were estimated as a function of the plasticizer mixtures with a full cubic mixture model^40^ (eq 5):

5 $$y=\overset{q}{\underset{i=1}{\sum }}\beta_{i}x_{i}+\sum \overset{q}{\underset{i<j=2}{\sum }}\beta_{ij}x_{i}x_{j}+\sum \overset{q}{\underset{i<j=2}{\sum }}\delta_{ij}x_{i}x_{j}\left(x_{i}-x_{j}\right)+\beta_{ijk}x_{i}x_{j}x_{k}+e$$

where *y* denotes an observed film property, *β* denotes the model coefficients, *x* denotes the relative proportions of the plasticizers in the mixture, and *e* denotes the model residual. The model coefficients β were determined by minimizing the sum of the squares of the model residuals using the least-squares estimate.^41^ The statistical significance of the coefficients was evaluated by comparing the variation explained by the model term against the model residuals using an *F* test. Statistically insignificant (*p* > 0.10) terms were then excluded from the models. The predictive ability of the models was determined with validation experiments. A combined validation error was determined as the root mean squared error (RMSE) (eq 6):

6 $$\mathrm{RMSE}=\sqrt{\frac{\overset{n}{\underset{i=1}{\sum }}{\left(y_{i}-{ŷ}_{i}\right)}^{2}}{n}}$$

where *y* and *ŷ* denote observed and predicted film properties, respectively, and *n* denotes the number of validation experiments. With response transformations, the predicted values were transformed back to their real units before determining the RMSE. The calculations were performed with MATLAB (MathWorks Inc.) and Design-Expert (Stat-Ease Inc.), and the results were plotted in OriginPro 2024 (OriginLab Corp.).

## Results and Discussion

### Technical Details

We studied plasticizer mixing to control the properties of regenerated cellulose films. Systematic variations in the experimental data were first determined with principal component analysis. The data are shown in Table S1 and the principal component loadings based on the first 13 experiments after data normalization are shown in Figure 1. Variables with a positive correlation were located in the same direction from the origo in the loadings plot, and the length of the loading vectors indicated the magnitude of the variable effects explained by the principal components. The results showed that ultimate tensile strength and Young’s modulus increased with increasing maltitol content in the plasticizer mixtures based on the first two principal components. Improved film strength was, however, associated with a decreased strain at break. We used the strain at break as an indication of film elasticity, which is an important property to prevent the film from breaking during handling and further processing. Water vapor and oxygen permeability of the films increased toward increasing glycerol in the mixtures (Figure 1a), as was expected based on the study by Fernández-Santos et al.^28^ Despite its nonbeneficial effects, we used glycerol to compare our results with those previously published in the field^18,23,42^ and to include plasticizers with a consistent range of hydroxyl group numbers (3, 6, and 9). These observations indicated that the barrier properties measured from glycerol-plasticized films improved with other plasticizers. The variable correlations explained by the first and the third principal components suggested that sorbitol also contributed to increased film strength (Figure 1b). Overall, the first three principal components explained 83% of the variation in the normalized data (Figure S1b) and provided a useful overview of the main correlations across the controlled plasticizer components and the determined film properties.

**Figure 1:**
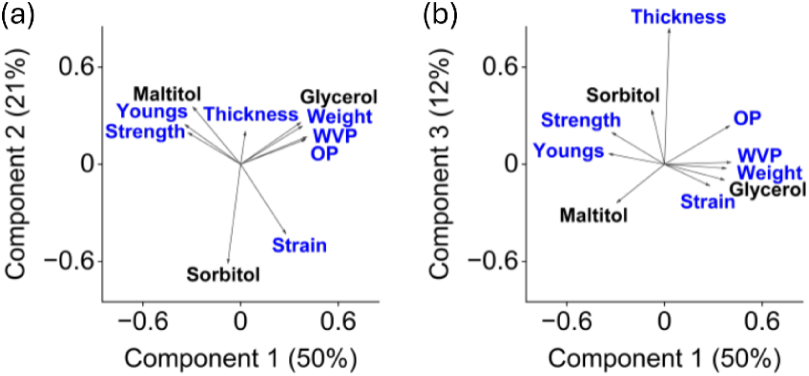
Principal component loadings based on the first and the second components in Figure a and the first and the third components in Figure b. Strength in the figure refers to ultimate tensile strength, Youngs refers to Young’s modulus, Weight referes to film weight per square meter, WVP referes to water vapor permeability, and OP referes to oxygen permeability.

We then used linear regression to estimate film properties based on the plasticizer mixture composition. We focused on tensile strength and water vapor and oxygen permeabilities as they are considered key indicators of film efficacy in barrier applications. Our hypothesis was that mixing low-molecular-weight polyols with a different number of hydroxyl groups would enable us to control these properties based on mixture composition. Regression model equations (eqs S1–S3) are shown in Supporting Information. Our tensile strength model equation showed clear indication of the synergistic effects between the individual plasticizers, but a similar interaction was not seen for water vapor or oxygen permeabilities. The final regression models are summarized in Table 1. The *R* ^2^ values indicated that the regression models explained 69–91% of the variation in the estimated film properties. The predictive ability of the models was evaluated with leave-one-out cross-validation. The resulting *R* ^2^_cv_ values showed that the predictions covered 49–84% of the variation in the predicted film properties (Table 1). These results indicated that especially the models for ultimate tensile strength and water vapor permeability provided a good fit to the data and could potentially provide satisfactory predictions within the experimental design range. Our model for oxygen permeability showed larger uncertainties, likely due to film heterogeneity. The dissolved cellulose dopes were not filtered before casting, which could have resulted in some undissolved cellulose fractions in the final films. We were unsuccessful in building a regression model for strain at break, likely due to large uncertainties in the data.

**Table 1:** Summary of the Regression Models on Film Properties

Film property	Unit	Transformation	Residual degrees of freedom	*R* ^2^	*R* ^2^_cv_	RMSE^a^	Range error ratio^b^
Ultimate tensile strength	MPa	None	8	0.91	0.73	7.5	2.1
Water vapor permeability	g·μm· m^–2^·day^–1^	log10	10	0.90	0.84	117.9	3.0
Oxygen permeability	cc·μm·m^–2^·d^–1^	log10	10	0.69	0.49	57.4	1.7

a Determined based on separate validation experiments.

b Determined based on the validation results and the RMSE.

Figure 2a shows a visualization of the experimental design. Examples of measured film properties and the measured vs predicted film properties based on the tensile strength model are shown in Figure 2b,c and the corresponding results for water vapor and oxygen permeability are provided in Figure S2. Response surfaces of the model predictions as a function of the plasticizer mixture composition are illustrated in Figure 2d. Based on the results, the highest measured strength values were determined when maltitol was used as a plasticizer, as suggested by the principal components (Figure 2b). Higher-than-average strength values were also determined from the ternary mixtures, where glycerol was used as the majority component (Figure 2b,d). Films treated with glycerol and sorbitol mixtures showed the highest strain-at-break values of 17% compared with the other plasticizer mixtures (Table S1). Our observations on the effects of individual plasticizers were partly in line with those previously reported for cellulose nanocrystal films by Fernández-Santos et al.^28^ The authors studied the effects of individual plasticizers and the plasticizer dose on film properties and reported higher strength values for maltitol than for glycerol at higher concentrations. Their cellulose nanocrystal films showed comparable elongation-at-break values with 10% glycerol and maltitol, but maltitol led to considerably higher elasticities when the plasticizer dose was increased to 25%.^28^ These observations were not supported by our results for regenerated cellulose films, which suggested that an increasing number of hydroxyl groups in the 5% plasticizer solution improved film strength but made the films more brittle. Our data, however, showed considerable variations in the strain-at-break values from the replicate experiments. These variations were likely generated by inconsistencies in film thickness and quality, which led to an uncontrolled break during strain. Our control films showed ultimate tensile strength and strain at break values of 62.3–74.3 MPa and 5.1–5.5% (*n* > 6), respectively, whereas the plasticized films showed on average a 41.0 MPa ultimate tensile strength and 12.1% strain at break. This indicated that plasticization generally weakened film strength and improved film elasticity as was expected.

**Figure 2:**
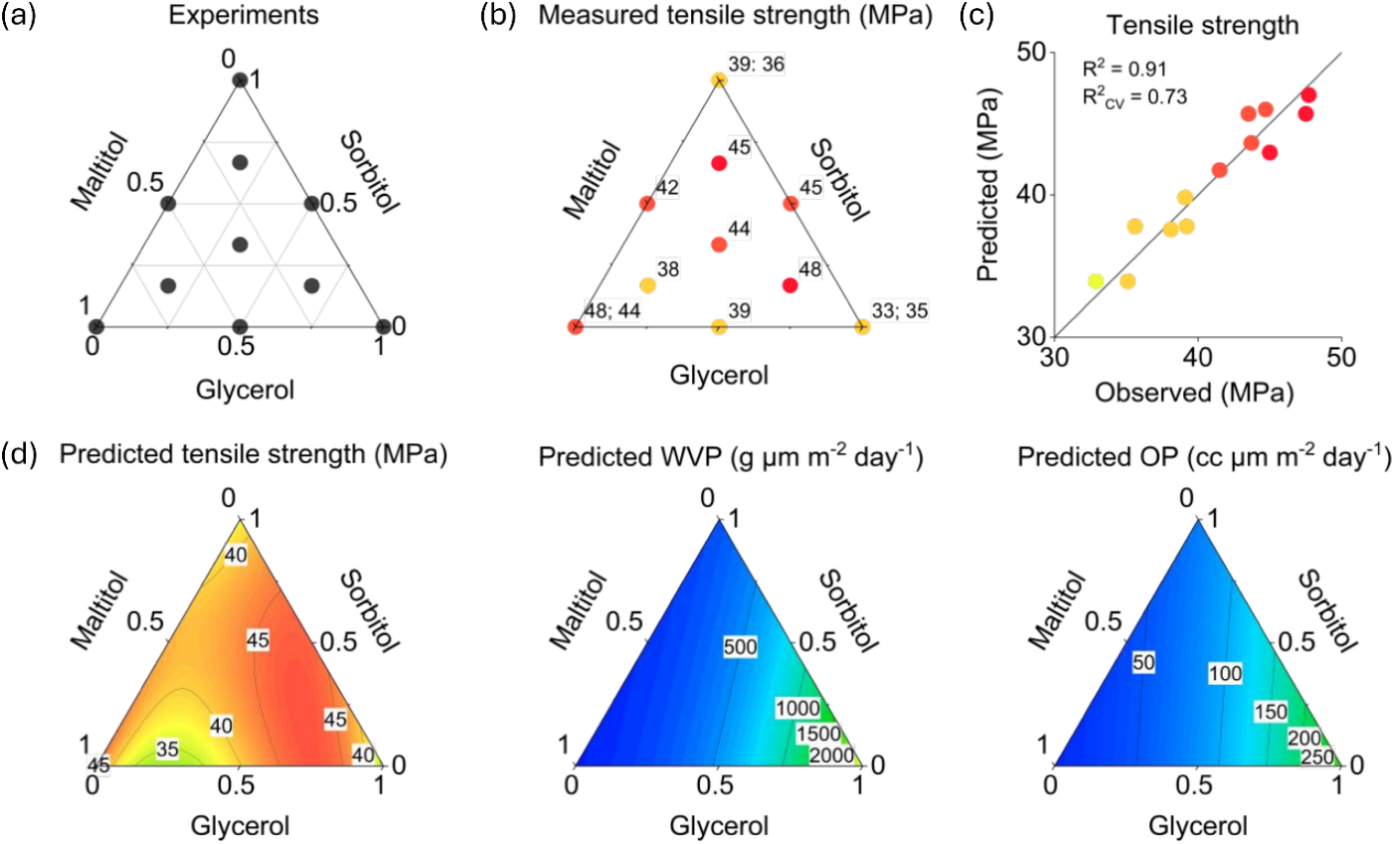
A visualization of the plasticizer mixture experiments corresponding to the plasticizer composition in the treatment solution (a), measured strength values from the experiments (b), measured vs predicted strength values based on the regression model (c), and the model predictions for ultimate tensile strength, water vapor permeability (WVP), and oxygen permeability (OP) (d).

Water vapor and oxygen permeability are key indicators of film efficacy for barrier packaging applications. One major disadvantage of cellulose-based films is their sensitivity toward water. Glycerol has been widely used for plasticizing cellulose films^18^ but is known to increase water vapor and oxygen permeability compared to nonplasticized films.^28,29^ The principal components showed that the barrier performance of the films deteriorated toward increasing glycerol content in the plasticizer mixtures (Figure 1a). The predictions of the individual regression models for water vapor and oxygen permeability are illustrated in Figure 2d. These results showed that the permeabilities decreased considerably with increasing sorbitol and maltitol content in the plasticizer mixtures. Especially, maltitol was effective in improving the barrier performance of the films and decreased the average measured water vapor and oxygen permeabilities by 93–94% compared to the use of glycerol (Figure S2). We also determined the water vapor and oxygen permeabilities of uncoated cellulose films produced by the viscose process at 23 °C and 50% relative humidity for comparison. These commercial films showed mean water vapor and oxygen permeabilities of 2640 g·μm·m^2^·day^–1^ and 480 cc·μm·m^2^·day^–1^, respectively. These results indicated that our regenerated cellulose films had considerably better barrier properties than the uncoated cellulose films currently on the market.

The molecule diffusion rate and cellulose interaction with the gas are known to be the most dominant factors in gas permeation, while porosity also has its contribution to the phenomena.^28,43,44^ Due to complete solubilization–precipitation, pores were not expected to play a vital role for the permeability of our regenerated cellulose films. Bharadwaj used a tortuosity-based model to describe the molecule permeability in filled polymers and highlighted that the polymer–filler interaction, state of aggregation, and especially the relative orientation of the filler within the polymer matrix significantly improve the barrier properties.^45^ Similarly, Fernández-Santos et al. concluded that incorporation of biodegradable additive could limit water vapor and oxygen permeabilities by complicating the network for the molecules to pass through due to increased tortuosity.^28^ In our study, plasticizers may have served as nucleation agents inducing crystallinity in the matrix or interact with the polymer matrix by forming hydrogen bonds and intercross-linking through opposite charges and thus potentially enhanced film gas barrier.

Plasticizer mixture composition had a considerable effect on the barrier properties of the films. The plasticization of regenerated cellulose films is generally described through hydrogen bond formation between the hydroxyl groups of the plasticizer and those of the cellulose polymer matrix.^46^ We hypothesized that the number of hydroxyl groups in the plasticizer solution might also have influenced film crystallinity,^28^ which likely contributed to the barrier and tensile performance of the films.^47^ A moist, never-dried, regenerated cellulose film can be described as a swelled web of cellulose polymer chains, and low molecular weight polyols can be expected to easily diffuse between these chains. At room temperature, sorbitol and maltitol are both solid compounds with high crystallinity. We anticipated that this plasticizer crystallization could also be seen as an increase in the crystallinity index of the dried films. Thus, we determined the total crystallinity indices of the films with wide-angle X-ray scattering. The scatterings and crystallinity results are shown in Figure 3a,b. Distinctive peaks at locations I_110_, I_020_, and I_004_ confirmed the cellulose II allomorph (Figure 3a),^48^ which generally forms during the precipitation of dissolved cellulose.^49^ We did not observe distinct peaks at around 12° which are usually indicative for hydrogen bond at crystalline plane I_1–10_ originating from either cellulose II or its hydrate.^50^ We, however, observed a peak at around 28° which could be part of the crystal structure of cellulose II. We observed considerable similarities in our diffraction patterns and in those from previous studies,^51,52^ suggesting that the unidentified peak did not originate from the used polyhydroxylic compounds, but was likely part of cellulose II or originated from our measurement geometry.

**Figure 3:**
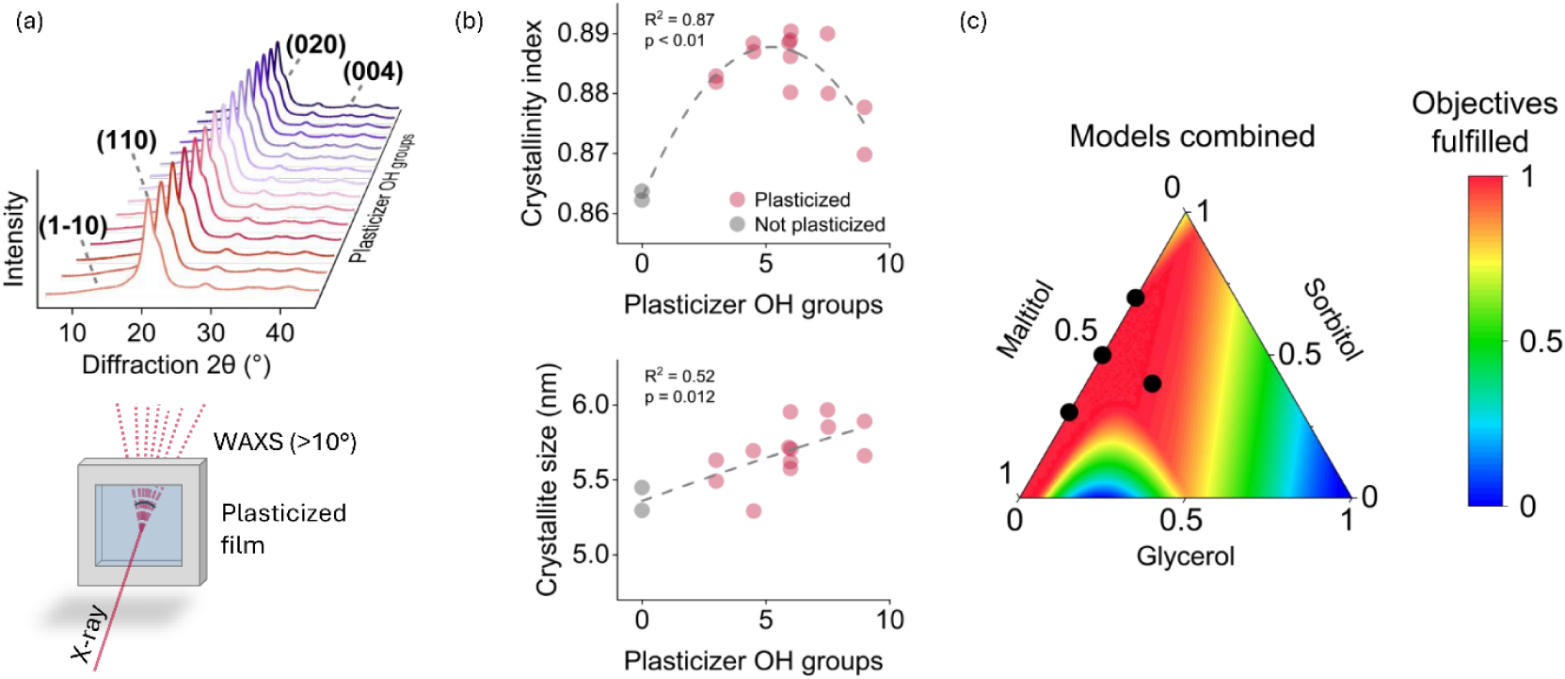
Schematic illustration of the wide-angle X-ray scattering (WAXS) measurement and diffraction patterns of plasticized regenerated cellulose films in (a), crystallinity indices and crystallite sizes of the main crystalline planes (I_110_) as a function of the hydroxyl groups present in the plasticizer mixture solutions (b), and the model predictions combined to achieve a tensile strength >40 MPa and water vapor and oxygen permeabilities <300 g·μm·m^–2^·day^–1^ and <75 cc·μm·m^–2^·day^–1^, respectively (c). The scatter symbols in Figure c show validation experiments. Plasticizer OH groups were calculated by multiplying the number of hydroxyl groups in a single plasticizer by the corresponding mass fraction in the mixture solution.

The plasticized films showed differences in the diffraction peak intensities at approximately 20° (Figure 3a). Thus, we used the Scherrer equation to determine crystallite size of the crystalline plane (I_110_) of the films.^37,38^ Exact fitting of peak I_110_ was difficult due to the overlapping peak I_020_. The determined crystallite sizes of our model films were in the range 5.29–5.97 nm, whereas the crystallite sizes of the nonplasticized controls were 5.30 and 5.45 nm. The increase in crystallite size can be explained by the crystallization of the polyols onto the cellulose surfaces or the crystallization of the polyols themselves. Our films plasticized with 50% sorbitol and 50% maltitol showed the largest crystallite size of 6.24 nm, which can be explained with different crystallization tendencies of the polyols or possible interactions between the different polyol plasticizers.

We note that determining the crystallinity index of regenerated cellulose accurately with the Segal equation is challenging, but the method can be used to determine trends between different films.^35^ Yamane et al.^53^ reported that a higher planar orientation of I_1–10_ and a higher crystallinity increased the density of polar hydroxyl groups on the surface of regenerated cellulose films, which resulted in higher wettability (i.e., higher hydrophilicity). Materials with high wettability tend to show higher water vapor permeability, because they can absorb and transmit more polar water molecules through their structure. Our films with the highest crystallinities (Figure 3b), however, did not result in the lowest water vapor permeabilities (Figure 2d). Our results shown in Figure 3b suggested that film crystallinity followed a quadratic relationship with the number of hydroxyl groups in the plasticizers (*R* ^2^ = 0.87, *p* < 0.01). The total crystallinity indeces for our plasticized films were in the range of 0.87–0.89, whereas the nonplasticized control films showed a total crystallinity index of 0.86. The highest crystallinities were observed with films that were plasticized with mixtures containing 5–8 hydroxyl groups and both crystallinity and water vapor permeability decreased when pure maltitol solution was used as the plasticizer. These observations suggested that an increase in the number of hydroxyl groups in the plasticizer mixture did not necessarily lead to an increase in the hydroxyl groups settled parallelly on the surface of the films, which would likely increase water transport.

High crystallinity influences mechanical properties by providing greater resistance to polymer deformation because highly crystalline materials have more ordered and tightly packed domains. Amorphous domains in cellulose materials are less ordered and provide weaker points for the material to fracture but are also more flexible.^54–56^ The lowest tensile strengths (32.9 MPa) were obtained with films plasticized with pure glycerol solutions and mediocre crystallinities (Figure 3b), while nonplasticized films showed the highest tensile strengths (74.3 MPa) yet with the lowest total crystallinity indices. The highest tensile strengths in the plasticized films were obtained with sorbitol-maltitol solutions (54.2 and 49.2 MPa), which also showed the highest crystallinity indices. Müller-Dethlefs and Hobza showed that in addition to hydrogen bond interactions, the biomolecular structure and functions are regulated by the stacking interactions between the intra- and intersheets of cellulose layers. While the hydrogen bonds emerge from charge transfer between specific atoms, stacking is an influence of the combination of van der Waals interactions, π–π interactions, and hydrophobic forces.^57^ The improved tensile strengths of our sorbitol and maltitol plasticized films were likely due to newly formed hydrogen bonds and stacking interactions between cellulose and the plasticizers as reported by Liu et al.^58^

Maltitol is a relatively complex sugar alcohol compound with 9 hydroxyl groups and a pyranose structure attached to a linear carbon chain. It has a molar mass of 344 g·mol^–1^ that is nearly four times higher than the molar mass of glycerol (92 g·mol^–1^) and nearly two times higher than the molar mass of sorbitol (192 g·mol^–1^). The complex and large molecule structure of maltitol may have weakened its ability to penetrate and distribute evenly between the polymer chains of the cellulose matrix.^59^ Penetration of a larger molecule would likely result in higher swelling of the film.^46^ We measured the film thicknesses (Table S1) but were unable to identify clear and systematic trends in thickness as a function of the plasticizer bath composition. We also measured the film weights (Table S1) and calculated the plasticizer contents based on the relative shares of the plasticizers in the treatment solution. We assumed comparable diffusion rates of glycerol, sorbitol, and maltitol into the regenerated cellulose films during film plasticization. The plasticizer composition in the bath, however, does not necessarily reflect the ratio in the resulting film, as differences in compatibility and diffusivity can lead to selective absorption. Total plasticizer contents in the films were all in the range of 16–33 wt %. Films treated with pure glycerol, sorbitol and maltitol solutions had plasticizer contents of 29, 19, and 20 wt %, respectively. Minor differences in plasticizer contents were likely generated by differences in molecular size and could implicate a more thorough distribution of glycerol across the film structure compared to its larger sorbitol and maltitol references. Different plasticizer distributions may have resulted in different tortuosity pathways. Müller et al. reported in average 5.0 × 10^–8^ m^2^ h^–1^ and 1.6 × 10^–8^ m^2^ h^–1^ water vapor diffusion coefficients for starch films with 25–35 wt % of glycerol or sorbitol, respectively,^42^ indicating a significantly lower water vapor diffusion coefficient of sorbitol as its inherent property. Differing tortuosity pathway and sorbitol’s lower diffusion coefficient against water vapor altogether resulted in better water vapor barrier in our films. Thorough understanding of the mechanisms would, however, require further studies related to the distribution of the plastizers on the surface and across the film structure and especially detailed water interaction study on film surface (e.g., with quartz crystal microbalance method).

Film efficacy in barrier packaging applications is affected by both mechanical and barrier properties. We combined the predictions of our individual regression models to identify promising plasticizer mixture compositions for given film properties. We aimed at a tensile strength >40 MPa coupled with water vapor and oxygen permeability <300 g·μm·m^–2^·day^–1^ and <75 cc·μm·m^–2^·d^–1^, respectively. The individual model predictions were first normalized to the range ([0,1]) based on these objectives, and the normalized predictions were then multiplied by one another to identify plasticizer compositions where the objectives were fulfilled. The results are shown in Figure 3c and indicated that promising plasticizer mixture compositions were located in the proximity of binary sorbitol-maltitol mixtures. We then designed four additional validation experiments to empirically test the predictions of our regression models with these promising plasticizer mixtures. The validation experiments are visualized in Figure 3c. For example, cellulose films plasticized with 50% sorbitol and 50% maltitol solution showed a tensile strength of 49.7 MPa coupled with water vapor and oxygen permeabilities of 259 g·μm·m^–2^·day^–1^ and 28 cc·μm·m^–2^·d^–1^, respectively, which successfully met the set objectives. The rest of the validation results are given in Table S1. The resulting RMSE values in Table 1 show the average root-mean-square error during model validation. The subsequent range error ratios suggested that the model for water vapor permeability showed in comparison with the lowest validation errors, followed by the models for ultimate tensile strength and oxygen permeability.

### Practical Considerations

Our objective was to improve regenerated cellulose films by focusing on plasticizer mixing. The experimental results showed that mixing simple polyols could be used to control film strength and water vapor and oxygen permeabilities with acceptable reliability. These findings are relevant as they indicate that plasticizer mixing provides a tool to improve regenerated cellulose films, which are currently not considered plastics in the single-use plastics directive by the European Union.^60^ The United Nations Environmental Assembly recently convened an Intergovernmental Negotiating Committee to develop a legally binding resolution on global plastic pollution with the aim to reduce plastic production and single-use plastics and to promote plastic recycling,^61^ which will further increase the demand for plastic alternatives in the future. We compared our results on the barrier properties of regenerated cellulose films and commercial cellophane against conventional petroleum-based plastics reported by Lavoine et al.^62^ The results shown in Figure 4 and indicate that our films matched the barrier performance of commercial cellophane and with further improvements could provide a competitive renewable alternative to polyolefin films.

**Figure 4:**
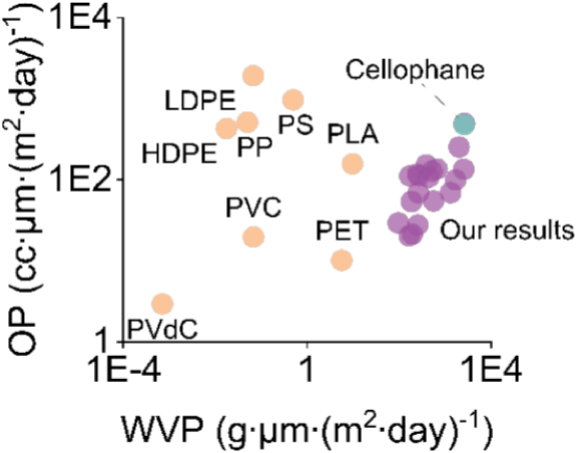
Our results on the barrier performance of regenerated cellulose films and commercial cellophane compared with conventional plastics reported by Lavoine et al.^62^.

We used mechano-enzymatic pretreatment and sodium zincate dissolution to prepare regenerated cellulose films. This approach enables producing films without the use of hazardous carbon disulfide and the generation of sulfur-containing byproducts, which are characteristic of regenerated cellulose production for viscose yarns. We, however, found no published data to assess the economics of our dissolution and precipitation approach; other than that, it could be potentially installed in existing viscose production plants.^63^ The global plastic film market size was 134.5 billion USD in 2023 and is expected to grow by 31% within the next five years. We anticipate that most of the process costs associated with large-scale film production would be generated by the dissolution and film preparation steps and that the price of the polyol mixtures for plasticization would be comparatively lower. ChemAnalyst reported that the price of glycerol fluctuated 2–6% within 2023–2024 due to supply constraints, rising production costs of palm oil, and geopolitical tensions.^64^ By the end of 2024, glycerol prices settled to 383–943 USD per metric tonne in Shanghai, Saudi Arabia, and South Korea.^65^ By the end of the quarter 2024, 70% sorbitol costed 731–1340 USD per metric tonne in New York, China, and France.^66^ Maltitol costed 1300–3600 USD per metric tonne in India, United Arab Emirates, Bangladesh, and Cameroon.^67^ Our findings on plasticizer mixing could potentially be applied also to other dissolution and precipitation chemistries. For example, *N*-methylmorpholine *N*-oxide dissolution is already in commercial use in the production of lyocell fibers for textiles and has shown acceptable chemical recovery rates and profitability when raw material and capital costs are reasonable.^68^ Overall, we foresee an increasing future demand for renewable and competitive alternatives to conventional plastics based on existing European Union regulations and the recent United Nations initiative.

## Conclusions

We studied the effects of glycerol, sorbitol, and maltitol on the properties of regenerated cellulose films based on plasticizer mixing. Our results showed that mixing the three polyols enabled us to control film strength and water vapor and oxygen permeabilities, which are key indicators for evaluating film efficacy in barrier packaging applications. Plasticizer mixing thus provides a novel and simple approach to improving the mechanical and barrier properties of regenerated cellulose films as an alternative to plastics in applications where higher elasticity is required. The barrier performance of our plasticized films was better than that of commercial uncoated cellophane film and they could be a potential renewable alternative to fossil-based polyolefin films with further improvements. These findings are important to combat the low recycling rates of conventional plastic films in packaging applications and to adapt to the current European Union regulations and the United Nations initiative to improve plastic recycling, reduce single-use plastics, and reduce the overall plastics production. Plasticization of the regenerated cellulose films will greatly benefit from property prediction with regression models toward targeted applications. We foresee an increasing demand for renewable and competitive alternatives for conventional plastics in packaging and other applications.

## Supplementary Material



## Notes

Film compositions and results data (Table S1); example of a stress–strain curve and its translation into data and a scree plot for principal component analysis (Figure S1a,b); regression model equations (eqs S1–S3); measured and the measured vs predicted water vapor and oxygen permeabilities (Figure S2) (PDF)

## References

[1] OECD. Global Plastics Outlook: Plastics use by application; OECD Environment Statistics (database). 10.1787/234a9f22-en.

[2] Hirvikorpi T., Vähä-Nissi M., Mustonen T., Iiskola E., Karppinen M. (2010). Atomic Layer Deposited Aluminum Oxide
Barrier Coatings
for Packaging Materials. Thin Solid Films.

[3] Mokwena K. K., Tang J. (2012). Ethylene Vinyl Alcohol: A Review of Barrier Properties for Packaging
Shelf Stable Foods. Crit. Rev. Food Sci. Nutr..

[4] Struller C. F., Kelly P. J., Copeland N. J. (2014). Aluminum Oxide Barrier Coatings on
Polymer Films for Food Packaging Applications. Surf. Coat. Technol..

[5] de
Mello Soares C. T., Ek M., Östmark E., Gällstedt M., Karlsson S. (2022). Recycling of Multi-Material Multilayer
Plastic Packaging: Current Trends and Future Scenarios. Resour., Conserv. Recycl..

[6] Ragaert K., Delva L., Van Geem K. (2017). Mechanical
and Chemical Recycling
of Solid Plastic Waste. Waste Manage..

[7] Geyer R., Jambeck J. R., Law K. L. (2017). Production
Use, and Fate of All Plastics
Ever Made. Sci. Adv..

[8] OECD. Global Plastics Outlook: plastic waste in 2019. OECD Environment Statistics (database). 10.1787/a92f5ea3-en.

[9] OECD. Global Plastics Outlook: plastic waste by end-of-life fate - projections; OECD Environment Statistics (database). 10.1787/3f85b1c2-en.

[10] Ragonnaud, G. EU Legislation in Progress - Revision of the Packaging and Packaging Waste Directive; European Parliament Research Service. 2023.

[11] Kolpak F.
J., Blackwell J. (1976). Determination
of the Structure of Cellulose II. Macromolecules.

[12] Singh, P. ; Duarte, H. ; Alves, L. ; Antunes, F. ; Le Moigne, N. ; Dormanns, J. ; Duchemin, B. ; Staiger, M. P. ; Medronho, B. From Cellulose Dissolution and Regeneration to Added Value Applications — Synergism Between Molecular Understanding and Material Development. In Cellulose - Fundamental Aspects and Current Trends. Poletto, M. ; Ornaghi, H. L., Jr. Eds.; IntechOpen, 2015, pp. 1–45.

[13] Klemm D., Heublein B., Fink H.-P., Bohn A. (2005). Cellulose: Fascinating
Biopolymer and Sustainable Raw Material. Angew.
Chem., Int. Ed..

[14] Grönqvist S., Hakala T. K., Kamppuri T., Vehviläinen M., Hänninen T., Liitiä T., Maloney T., Suurnäkki A. (2014). Fibre Porosity
Development of Dissolving Pulp during Mechanical and Enzymatic Processing. Cellulose.

[15] Grönqvist S., Kamppuri T., Maloney T., Vehviläinen M., Liitiä T., Suurnäkki A. (2015). Enhanced Pre-Treatment
of Cellulose
Pulp Prior to Dissolution into NaOH/ZnO. Cellulose.

[16] Vehviläinen M., Kamppuri T., Grönqvist S., Rissanen M., Maloney T., Honkanen M., Nousiainen P. (2015). Dissolution of Enzyme-Treated Cellulose
Using Freezing–Thawing Method and the Properties of Fibres
Regenerated from the Solution. Cellulose.

[17] Vehviläinen M., Määttänen M., Grönqvist S., Harlin A., Steiner M., Kunkel R. (2020). Sustainable Continuous
Process for Cellulosic Regenerated Fibers. Chem.
Fibers Int..

[18] Xiao C., Zhang Z., Zhang J., Lu Y., Zhang L. (2003). Properties
of Regenerated Cellulose Films Plasticized with α-Monoglycerides. J. Appl. Polym. Sci..

[19] Lee K. J., Choi K. (2024). Environmental Occurrence
Human Exposure, and Endocrine Disruption
of Di-Iso-Nonyl Phthalate and Di-Iso-Decyl Phthalate: A Systematic
Review. Crit. Rev. Environ. Sci. Technol..

[20] Vessa B., Perlman B., McGovern P. G., Morelli S. S. (2022). Endocrine Disruptors
and Female Fertility: A Review of Pesticide and Plasticizer Effects. F S Rep..

[21] Rebelo R. C., Ribeiro D. C. M., Pereira P., De Bon F., Coelho J. F. J., Serra A. C. (2023). Cellulose-Based Films with Internal
Plasticization
with Epoxidized Soybean Oil. Cellulose.

[22] Jin Z., Wang S., Wang J., Zhao M. (2012). Effects of Plasticization
Conditions on the Structures and Properties of Cellulose Packaging
Films from Ionic Liquid [BMIM]­Cl. J. Appl. Polym.
Sci..

[23] Peng J., Li Y., Liu X., Ke G., Song D., Wu S., Xu W., Zhu K. (2021). Cellulose Film with Air Barrier and Moisture-Conducting
Character Fabricated by NMMO. J. Mater. Sci.

[24] de
Britto D., de Rizzo J. S., Assis O. B. G. (2012). Effect of Carboxymethylcellulose
and Plasticizer Concentration on Wetting and Mechanical Properties
of Cashew Tree Gum-Based Films. Int. J. Polym.
Anal. Charact..

[25] Sanyang M. L., Sapuan S. M., Jawaid M., Ishak M. R., Sahari J. (2015). Effect of
Plasticizer Type and Concentration on Tensile, Thermal and Barrier
Properties of Biodegradable Films Based on Sugar Palm (Arenga Pinnata. Starch. Polymers.

[26] Cazón P., Velázquez G., Vázquez M. (2020). Regenerated Cellulose Films Combined
with Glycerol and Polyvinyl Alcohol: Effect of Moisture Content on
the Physical Properties. Food Hydrocoll..

[27] Zhang Y.-Q., Li J., Huang X.-J., Yang C.-X., Wu C., Yang Z.-L., Li D.-Q. (2023). Performance-Enhanced Regenerated
Cellulose Film by Adding Grape Seed
Extract. Int. J. Biol. Macromol..

[28] Fernández-Santos J., Valls C., Cusola O., Roncero M. B. (2021). Improving Filmogenic
and Barrier Properties of Nanocellulose Films by Addition of Biodegradable
Plasticizers. ACS Sustainable Chem. Eng..

[29] Moreira R., Rebelo R. C., Coelho J. F. J., Serra A. C. (2024). Novel Thermally
Regenerated Flexible Cellulose-Based Films. Eur. J. Wood Wood Prod..

[30] Scheffe H. (1963). The Simplex-Centroid
Design for Experiments with Mixtures. J. R.
Stat. Soc. Ser. B.

[31] ISO 5351:2010. Pulps — Determination of Limiting Viscosity Number in Cupri-Ethylenediamine (CED) Solution; 2010. https://standards.iteh.ai/catalog/standards/sist/cfd6c695-626a-4c82-9a21-aac7a023a09f/iso-5351-2010.

[32] McNaught, A. D. ; Wilkinson, A. Mark–Houwink Equation. In The IUPAC Compendium of Chemical Terminology; International Union of Pure and Applied Chemistry: Research Triangle Park; (IUPAC)NC, 2014.

[33] Park S., Baker J. O., Himmel M. E., Parilla P. A., Johnson D. K. (2010). Cellulose
Crystallinity Index: Measurement Techniques and Their Impact on Interpreting
Cellulase Performance. Biotechnol. Biofuels.

[34] Azubuike C. P., Rodríguez H., Okhamafe A. O., Rogers R. D. (2012). Physicochemical
Properties of Maize Cob Cellulose Powders Reconstituted from Ionic
Liquid Solution. Cellulose.

[35] Nam S., French A. D., Condon B. D., Concha M. (2016). Segal Crystallinity
Index Revisited by the Simulation of X-Ray Diffraction Patterns of
Cotton Cellulose Iβ and Cellulose II. Carbohydr. Polym..

[36] Segal L., Creely J. J., Martin A. E., Conrad C. M. (1959). An Empirical
Method
for Estimating the Degree of Crystallinity of Native Cellulose Using
the X-Ray Diffractometer. Text. Res. J..

[37] French A. D., Santiago Cintrón M. (2013). Cellulose Polymorphy, Crystallite
Size, and the Segal Crystallinity Index. Cellulose.

[38] Scherrer, P. Bestimmung Der Größe Und Der Inneren Struktur von Kolloidteilchen Mittels Röntgenstrahlen Nachrichten von der Gesellschaft der Wissenschaften zu Göttingen, Mathematisch-Physikalische Klasse EuDML 1918 98–100

[39] Bro R., Smilde A. K. (2014). Principal Component Analysis. Anal. Methods.

[40] Myers, R. H. ; Montgomery, D. C. ; Anderson-Cook, C. M. Response Surface Methodology: Process and Product Optimisation Using Designed Experiments; Wiley Series in Probability and Statistics; John Wiley & Sons, Inc., 2009; p 680.

[41] Mäkelä M. (2017). Experimental
Design and Response Surface Methodology in Energy Applications: A
Tutorial Review. Energy Convers. Manage..

[42] Müller C. M. O., Yamashita F., Laurindo J. B. (2008). Evaluation of the Effects of Glycerol
and Sorbitol Concentration and Water Activity on the Water Barrier
Properties of Cassava Starch Films through a Solubility Approach. Carbohydr. Polym..

[43] Nair S. S., Zhu J., Deng Y., Ragauskas A. J. (2014). High Performance Green Barriers Based
on Nanocellulose. Sustain. Chem. Process..

[44] Wang J., Gardner D. J., Stark N. M., Bousfield D. W., Tajvidi M., Cai Z. (2018). Moisture and Oxygen Barrier Properties
of Cellulose Nanomaterial-Based Films. ACS Sustainable
Chem. Eng..

[45] Bharadwaj R. K. (2001). Modeling
the Barrier Properties of Polymer-Layered Silicate Nanocomposites. Macromolecules.

[46] Bonifacio A., Bonetti L., Piantanida E., De Nardo L. (2023). Plasticizer Design
Strategies Enabling Advanced Applications of Cellulose Acetate. Eur. Polym. J..

[47] Krässig, H. A. Structure and Physico-Chemical Properties. In Cellulose: structure, Accessibility and Reactivity, Huglin, M. B. , Ed.; Gordon and Breach Science Publishers: Yverdon, 1993; pp. 150–166.

[48] French A. D. (2014). Idealized
Powder Diffraction Patterns for Cellulose Polymorphs. Cellulose.

[49] Langan P., Nishiyama Y., Chanzy H. (2001). X-Ray Structure of
Mercerized Cellulose
II at 1 Å Resolution. Biomacromolecules.

[50] Kobayashi K., Kimura S., Togawa E., Wada M. (2011). Crystal Transition
from Cellulose II Hydrate to Cellulose II. Carbohydr.
Polym..

[51] Ahokas P., Mäkelä M., Jaiswal A., Khakalo A., Harlin A. (2024). Controlling
the Rheology of Cellulose Dissolved in 4 – Methylmorpholine,
N. – Oxide and Tensile Properties of Precipitated Cellulose
Films via Mixture Design. Cellulose.

[52] González
Carmona E., Schlapp-Hackl I., Jääskeläinen S., Järvinen M., Nieminen K., Sawada D., Hummel M., Sixta H. (2023). Development of Cellulose Films by Means of the Ioncell® Technology,
as an Alternative to Commercial Films. Cellulose.

[53] Yamane C., Aoyagi T., Ago M., Sato K., Okajima K., Takahashi T. (2006). Two Different Surface Properties of Regenerated Cellulose
Due to Structural Anisotropy. Polym. J..

[54] Chivers R. A., Moore D. R. (1994). The Effect of Molecular Weight and Crystallinity on
the Mechanical Properties of Injection Moulded Poly­(Aryl-Ether-Ether-Ketone)
Resin. Polymer.

[55] Bin Y., Oishi K., Yoshida K., Matsuo M. (2004). Mechanical Properties
of Poly­(Ethylene Terephthalate) Estimated in Terms of Orientation
Distribution of Crystallites and Amorphous Chain Segments under Simultaneous
Biaxially Stretching. Polym. J..

[56] Hahary F. N., Husseinsyah S., Zakaria M. M. (2016). Improved Properties of Coconut Shell
Regenerated Cellulose Biocomposite Films Using Butyl Methacrylate. BioResources.

[57] Müller-Dethlefs K., Hobza P. (2000). Noncovalent Interactions:
A Challenge for Experiment and Theory. Chem.
Rev..

[58] Liu X., Pang J., Zhang X., Wu Y., Sun R. (2013). Regenerated
Cellulose Film with Enhanced Tensile Strength Prepared with Ionic
Liquid 1-Ethyl-3-Methylimidazolium Acetate (EMIMAc). Cellulose.

[59] Ioelovich M. (2009). Accessibility
and Crystallinity of Cellulose. BioResources.

[60] European Parliament. The Reduction of the Impact of Certain Plastic Products on the Environment; European Union, 2019. https://eur-lex.europa.eu/eli/dir/2019/904/oj.

[61] UNEP. Resolution 5/14. End Plastic Pollution: Towards an International Legally Binding Instrument; United Nations Environment Programme. 2022, 2, 1–6.

[62] Lavoine N., Desloges I., Dufresne A., Bras J. (2012). Microfibrillated
Cellulose
- Its Barrier Properties and Applications in Cellulosic Materials:
A Review. Carbohydr. Polym..

[63] Heikkilä, P. ; Fontell, P. ; Kamppuri, T. ; Mensonen, A. ; Määttänen, M. ; Pitkänen, M. ; Raudaskoski, A. ; Vehmas, K. ; Vehviläinen, M. ; Harlin, A. Relooping Fashion Initiative; 2018. https://cris.vtt.fi/en/publications/the-relooping-fashion-initiative.

[64] ChemAnalyst. Polyol Price Trend and Forecast. Chemical Prices. https://www.chemanalyst.com/Pricing-data/polyols-60.

[65] ChemAnalyst. Glycerine Price Trend and Forecast. Chemical Prices. https://www.chemanalyst.com/Pricing-data/glycerine-1168.

[66] ChemAnalyst. Sorbitol Price Trend and Forecast. Chemical Prices. https://www.chemanalyst.com/Pricing-data/sorbitol-1274.

[67] PharmaCompass. Maltitol, API Reference Price, Table View. https://www.pharmacompass.com/price/maltitol.

[68] Hytönen E., Sorsamäki L., Kolehmainen E., Sturm M., von Weymarn N. (2023). Lyocell Fibre
Production Using NMMO – A Simulation-Based Techno-Economic
Analysis. BioResources.

