# Supplementary material for: Plasticizer Mixing Improved Regenerated Cellulose Films as an Alternative to Plastics
Source: ACS Sustain Chem Eng. 2025 Jul 7;13(28):10771–9. doi: 10.1021/acssuschemeng.5c00491 (PMC12309247; doi:10.1021/acssuschemeng.5c00491)
Supplement: Supplementary file 1 [file sc5c00491_si_001.pdf]

Supporting Information for

Plasticizer mixing improved regenerated cellulose films as an alternative to plastics

Pauliina Ahokas*, Vesa Kunnari, Johanna Majoinen, Ali Harlin and Mikko Mäkelä

VTT Technical Research Centre of Finland Ltd., Tietotie 4E, 02044 Espoo, Finland

*Corresponding author, e-mail: pauliina.ahokas@vtt.fi

Number of pages: 4

Number of figures: 2

Number of tables: 1

Table S1. Film results. Experiments 1-13 were used for determining the regression models and experiments 14-17 were used for model validation. The exact numerical values have been rounded.

Exp.	Plasticizer bath composition			Thickness (µm)	Weight (g·m^{-2})	Strength (MPa)	Strain at break (%)	Young's modulus (GPa)	WVP (g·µm·(m^2·day)$^{-1}$)	OP (cc·µm·(m^2·day)$^{-1}$)
	Glycerol	Sorbitol	Maltitol							
1	1	0	0	33	49	32.9	15.0	1.7	2669	494
2	0	1	0	32	39	39.2	13.7	2.2	399	153
3	0	0	1	30	40	47.5	3.8	3.4	97	29
4	0.5	0.5	0	29	44	44.7	17.1	2.5	1340	69
5	0.5	0	0.5	28	42	39.1	14.9	2.3	571	55
6	0	0.5	0.5	32	41	41.5	8.5	2.8	196	21
7	0.67	0.17	0.17	32	44	47.7	8.8	2.9	676	136
8	0.17	0.67	0.17	30	40	45.0	11.1	2.8	189	54
9	0.17	0.17	0.67	29	44	38.1	9.6	2.1	277	112
10	0.33	0.33	0.33	31	43	43.7	15.4	2.0	442	107
11	1	0	0	28	44	35.1	12.7	2.3	2044	253
12	0	1	0	28	42	35.6	16.1	2.2	267	69
13	0	0	1	31	41	43.5	11.1	2.5	168	20
14	0	0.5	0.5	30	42	54.2	11.5	3.1	174	111
15	0	0.3	0.7	29	40	49.7	10.6	2.5	259	28
16	0	0.7	0.3	30	42	38.5	17.0	1.9	256	117
17	0.2	0.4	0.4	28	40	40.9	13.8	2.3	528	128
Control	0	0	0	25	32	74.3	5.1	4.9	1713	99
Control	0	0	0	26	33	62.3	5.5	3.9	2601	134

Strength in the table refers to ultimate tensile strength, WVP to water vapour permeability and OP to oxygen permeability.

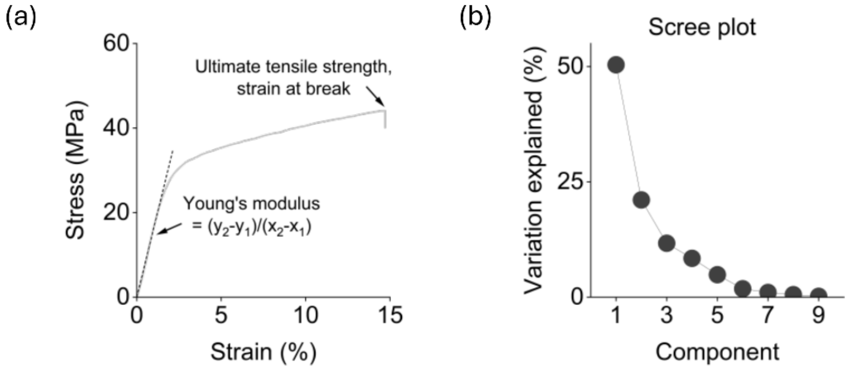

Figure S1. A typical stress-strain curve of a regenerated cellulose film as an example of how different tensile property parameters have been derived from the curve in (a), and a line plot of the variation explained by the different factors for principal component analysis in (b).

Model equations for the ultimate tensile strength, water vapour and oxygen permeabilities, respectively, Eq. (S1-S3):

$$y_1 = 33.92x_1 + 37.79x_2 + 45.69x_3 + 40.58x_1x_2 + 107.58x_1x_3(x_1 - x_3) \quad \text{(S1)}$$

$$y_2 = 10^{(3.35x_1+2.47x_2+2.10x_3)} \quad \text{(S2)}$$

$$y_2 = 10^{(2.45x_1+1.86x_2+1.40x_3)} \quad \text{(S3)}$$

where y_1, y_2 *and* y_3 denoted observed film properties and x_1, x_2 and x_3 the relative proportion of glycerol, sorbitol and maltitol, respectively, in the mixture solutions.

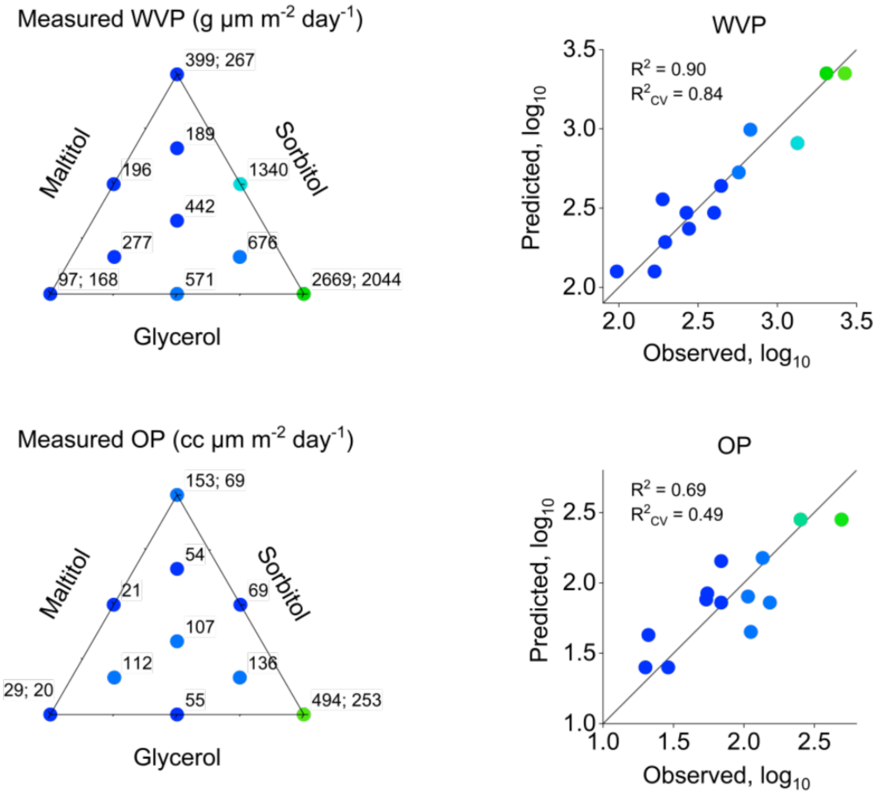

Figure S2. Measured and the measured vs. predicted water vapour permeability (WVP) values after log10 transformation (top row), and measured and the measured vs. predicted oxygen permeability (OP) values after log10 transformation (bottom row).
